# NETosis and the Immune System in COVID-19: Mechanisms and Potential Treatments

**DOI:** 10.3389/fphar.2021.708302

**Published:** 2021-08-05

**Authors:** Constant Gillot, Julien Favresse, François Mullier, Thomas Lecompte, Jean-Michel Dogné, Jonathan Douxfils

**Affiliations:** ^1^ Department of Pharmacy, University of Namur, Namur Research Institute for Life Sciences, Namur Thrombosis and Hemostasis Center, Namur, Belgium; ^2^ Department of Laboratory Medicine, Clinique St-Luc Bouge, Namur, Belgium; ^3^ Laboratory Hematology, Université Catholique de Louvain, CHU UCL Namur, Namur Research Institute for Life Sciences (NARILIS), Namur Thrombosis and Haemostasis Centre (NTHC), Yvoir, Belgium; ^4^ Division of Angiology and Haemostasis, University Hospitals of Geneva, Geneva, Switzerland; ^5^ Qualiblood s.a., Namur, Belgium

**Keywords:** COVID-19, SARS-CoV-2, NETosis, therapeutics, inflammation

## Abstract

NETosis is a form of neutrophil death leading to the release of extracellular chromatin and the assembling of proteins, including antiviral proteins, primed by an initial pathogenic stimulus. Under certain specific conditions, neutrophils can exhibit a double-edged activity. This event has been implicated in COVID-19 among other conditions. Neutrophil extracellular traps (NETs) are involved in the pathogenesis of COVID-19 by promoting a pro-inflammatory and a procoagulant state leading to multiorgan failure. This particular form of host defense promoted by neutrophils is closely related to the well-known cytokine storm observed in severe COVID-19 patients. These two elements therefore represent possible targets for treatment of severe SARS-CoV-2 infections.

## Introduction

The Severe Acute Respiratory Syndrome Coronavirus 2 (SARS-CoV-2) is responsible for the ongoing pandemic and is associated with significant morbidity and mortality. The number of confirmed cases approaches 194 million and the number of deaths worldwide has reached the four million mark. The most affected continents were America and Europe. Coronaviruses (CoVs) are single-stranded RNA enveloped viruses. They belong to the subfamily *Coronaviridae*. The HKU1, the NL63, the OC43 and the 229E are four common endemic coronaviruses causing diseases of low intensity. These four coronaviruses are called non-severe acute respiratory syndrome (SARS)-like coronaviruses (Coronaviridae Study Group of the International Committee on Taxonomy of Viruses, 2020). Three highly pathogenic coronaviruses have appeared. The first, which has become pandemic, was the SARS-CoV-1 (initially called “SARS-CoV”) reported in November 2002, followed by the Middle East respiratory syndrome CoV (the MERS-CoV) a decade later in June 2012. These two pandemics are over now (Coronaviridae Study Group of the International Committee on Taxonomy of Viruses, 2020). Lastly, the SARS-CoV-2 was identified in December 2019 in Wuhan, China. There are currently four variants of concern of the original SARS-CoV-2 strain called Alpha (B1.1.7) lineage United Kingdom, Beta (B.1.351) lineage South Africa, Gamma (P.1) lineage Brazil and Delta (B.1.617.2) lineage India (Forster et al., 2020; Yadav et al., 2021). Other variants will certainly emerge following a Darwinian evolution till full, ideally worldwide, protection afforded with massive vaccination.

This virus is mainly transmitted via the respiratory tract and is airborne via microdroplets. SARS-CoV-2 has the capacity to infiltrate the lower respiratory tract and generate a series of respiratory and systemic complications (Santesmasses et al., 2020). Older people, men and people with predisposing factors such as hypertension, diabetes, heart disease or cancer are at increased risk of developing complications from SARS-CoV-2 infection than other individuals (Marshall et al., 2020). The SARS-CoV-2 infection profile is complex due to its non-specific nature. Indeed, the symptoms are similar to many viral infections, which makes diagnosis complicated (Mackman et al., 2020). Patients suffering from coronavirus-disease-2019 (COVID-19) may be asymptomatic or may develop a mild, moderate or severe form of the disease (Marshall et al., 2020). Severe COVID-19 cases are characterized by an increase rate of lung infection, high serum levels of cytokines and an extensive lung damage with thrombosis in the microvasculature (Chen et al., 2020; Wang et al., 2020; Zhou et al., 2020). In severely affected patients, a pro-inflammatory and prothrombotic state is present (Wang et al., 2020). While the occurrence of the cytokine storm in severe COVID-19 cases is evident and has been identified by laboratory measurements, we do not know yet exactly what propagates and triggers the storm. An exacerbate host response in severe cases with the aberrant activation of neutrophils has been proposed as potential explanation (Wang et al., 2020). Indeed, an elevated neutrophil blood count predict poor outcomes in COVID-19 patients, and the neutrophil-to-lymphocyte ratio is known as a risk factor for severe COVID-19 (Liu et al., 2020).

One of the current challenges is to estimate the percentage of asymptomatic subjects who may unknowingly carry and transmit the virus. As the pandemic is currently worldwide, it will be unrealistic to eradicate the virus, especially because of the non-specific nature of the disease and the rapid emergence of variants which can in some way escape the immune response induced by a previous strain or the current vaccines available (Wang et al., 2021). Beside the hopes of vaccine efficacy for reducing the spread of COVID-19 and its severity, a thorough knowledge of the underlying physiopathological mechanisms of the disease is of upmost importance for the development of the most optimal treatments.

### Literature Search

We performed a review of the literature about NETs in COVID-19, by doing an electronic search on PubMed. Other search engines have not been consulted. We used the following keywords: “SARS-COV-2”; “COVID-19”; “COVID”; “COVID19” in combination with “NETosis”; “neutrophil extracellular traps”; “NETs”. Two researchers (CG and JD) independently screened all titles and abstracts identified from the literature search to determine potentially eligible manuscripts. After a first screening, articles were categorized as follows: “Review”; *In vitro* investigations”; “Ex vivo investigations”; “*In vivo* investigations”; “Clinical trials”. One article may be included in different categories. Only a subset of the papers is cited due to space restriction in the citation list and redundant information, especially for the reviews. Nevertheless, they can all be found in the Supplemental Material. (Supplemental Figure S1, Supplemental Data 1).

### Mechanisms of SARS-CoV-2

#### From Neutrophils to Neutrophil Extracellular Traps

Cells involved in innate immunity represent the first line defense of the body against an infective pathogen. Their role is to neutralize these pathogens and to trigger adaptive immunity in case of persistent infection. Given the growing evidence of their role in the antiviral response, neutrophils could be considered as the main “foot soldiers” of innate immunity in association with other blood elements such as platelets (Thierry and Roch, 2020). Neutrophils account for about 70% of circulating human leukocytes. They were known to have two main types of action, degranulation and phagocytosis. NETosis (Neutrophil Extracellular Traps formation) was also described as an additional mechanism of action (de Bont et al., 2019).

NETosis has also been described in the pathophysiology of viral infections other than COVID-19.

In normal conditions, NETosis is a regulated form of neutrophil death, which participates in the host’s immune defenses by the formation of traps to prevent the pathogen from spreading in the organism. This host defense mechanism has been reported in many infectious diseases such as infections by the respiratory syncytial virus (RSV), human immunodeficiency virus (HIV) or Chikungunya virus (CHIKV) (Brinkmann et al., 2004; Yu et al., 2018; de Bont et al., 2019). The stimulus that leads to the generation of neutrophils extracellular traps (NETs) include the presence of pathogen-associated molecular patterns (PAMPs) which are recognized by pattern recognition receptors (PRR) such as Toll-like receptor (TLR) 4, 7 or 8 (Hiroki et al., 2019), pro-inflammatory cytokines (interleukine-1β (IL-1β), IL-6, C-X-C chemokine 8 (CXCL-8), Tumor Necrosis Factor ⍺ (TNF-⍺)), activated platelets and the complement system via C3, CR1 or C5a (Thierry and Roch, 2020). The early onset of an inflammatory reaction associated with an increase local or general vascular permeability is a classic feature of acute viral infections such as SARS-CoV-2 (Thierry and Roch, 2020). During this NETosis, the neutrophils will release their chromatin via decondensation by action on histones. This decondensation is related to the activity of the enzyme peptidylarginine deiminase 4 (PAD4), which catalyzes the conversion of histone arginine to citrulline; the product is called citrullined-histone (Cit-H) (Yost et al., 2016). NETs formation is also facilitated by Gasdermin G, a protein which aims at generating pores into neutrophil membrane, allowing the rupture of the cell membrane and the liberation of the chromatin (Wen et al., 2009). Released chromatin is accompanied by several antibacterial proteins such as myeloperoxidase (MPO), neutrophil elastase (NE) or histones (de Bont et al., 2019; Thulborn et al., 2019). These proteins serve as scaffolding for the NETs but also give its antimicrobial properties, the most abundant being histones (de Bont et al., 2019). NE appears to be extremely important for the formation of NETs because it acts on chromatin decondensation, in the same way as PAD4, but it also acts on changes occurring at the histones level (Papayannopoulos et al., 2010). This process is lethal for neutrophils but in parallel to this “suicidal NETosis” there is a “vital NETosis” which does not involve the same vectors (Papayannopoulos et al., 2010).

This pathway is generally induced by bacterial stimulation and does not involve nicotinamide adenine dinucleotide phosphate (NADPH) oxidase and the formation of reactive oxygen species (ROS), unlike the suicide pathway. This response is also faster because it occurs within 30 min compared to 3 h for the suicide pathway (de Bont et al., 2019). ROS have the ability to increase the amount of myeloid suppressor cells (MDSCs), a sub-population of immature neutrophils (Yamamoto et al., 2018). However, no matter which pathway is involved, NETs are made up of chromatin associated with antibacterial proteins and are capable of interacting with the complement and with the coagulation system (Rohrbach et al., 2012; Sollberger et al., 2018; de Bont et al., 2019).

An important point is the clearance of NETs because many pathological conditions, especially vascular disorders leading to the occlusion of micro-vessels, can occur due to NET clearance deficiency. NETs are usually degraded by plasma DNases (DNase 1 and DNase 3) and are then eliminated by macrophages.

In fact, NETs have double-edged-sword activities. They are not only involved in the response to viral infections since they can be found in certain diseases where their presence is a sign of an inadequate immune response, leading to tissue damage. These include non-infectious inflammatory diseases, autoimmune diseases and other non-autoimmune diseases (Yu et al., 2018; Thierry and Roch, 2020). The most documented NET-related disease is systemic lupus erythematosus in which failure in NETs clearance has been observed. In SLE patients, NETs expose numerous autoantigens leading to this autoimmune reaction. The presence of excess NETs in these patients is detectable in the circulation but also at the tissue level. These NETs are furthermore resistant to DNases, which maintains an inflammatory state, leading to the maintenance of cytokine storm. All these factors generate the production of an amplification loop, which maintains the inappropriate autoimmune response (Thierry and Roch, 2020).

NETs can also trigger the formation of an inflammatory cascade leading to cancer metastasis, tissues damaging or multiple organ dysfunctions which are more often observed in the pulmonary, cardiovascular and renal systems (Bonow et al., 2020).

#### Inflammatory System and COVID-19

The understanding of the pathophysiology of COVID-19 remains incomplete, particularly in regard to the multi-organ failure it may cause. When the virus is detected in the lower respiratory tract, the immune machinery is set up with the activation of innate immunity (Thierry and Roch, 2020; Urwyler et al., 2020). Symptom aggravation can sometimes be abrupt in SARS-CoV-2 infections, and studies suggest that SARS-CoV-2, in addition to neutrophils activation as mentioned above, would also activate macrophages, T cells, natural killer (NK) cells, epithelial and endothelial cells to finally lead to a “cytokine storm” (Azkur et al., 2020; Kritas et al., 2020; Sun et al., 2020). The mast cells are activated by the virus patterns and will release a series of pro-inflammatory cytokines and chemokines. In a normal situation, mast cells fight the infection by attacking the pathogen directly. However, an excessive activation of these cells can lead to a hyper-inflammatory reaction, which is called “cytokine storm” (Kempuraj et al., 2016; Kempuraj et al., 2017). This cytokine storm itself leads to multi-organ failure which may be fatal (Thierry and Roch, 2020). Virus can also activate mast cells through TLRs and then increase the inflammatory mediator expression. Mast cells are also able to detect damage-associated molecular patterns (DAMPs) from SARS-CoV-2, triggering their respond against the virus (Vardhana and Wolchok, 2020). The cytokine storm is characterized by increased plasma levels of IL-1β, IL-2, IL-6, IL-7, CXCL-8, IL-10, IL-12, IL-17, gamma interferon (IFN-γ), Granulocyte-Colony-Stimulating-Factor (G-CSF), TNF-⍺, Chemokine (C-C motif) ligand 2 (CCL2), and CCL5 (Huang et al., 2020; Mehta et al., 2020; Ruan et al., 2020; Zhang et al., 2020). Increased expression of pro-inflammatory cytokines may be due to the action of Janus Activated Kinase (JAK) enzymes that regulate gene transcription through the phosphorylation of seven Signal Transducer and Activator of Transcription (STAT) factors (STAT-1/2/3/4/5A/5B/6), with consequent T-cell activation and cytokine release from immune cells, including IL-2, IL-6, IL-7, IL-12, IL-15, IL-21, IL-22, IL-23, and IFN-γ.

Lymphopenia has also been reported in patients with COVID-19 with a decrease in total T-cells, CD4^+^ T-cells, CD8^+^ T-cells and with an increase in T-helper 17 (Th17) proinflammatory cells (Pedersen and Ho, 2020). Indeed, increased IL-6 levels in patients suffering from a SARS-CoV-2 infection can induce Th17 cell differentiation, which would lead to deregulation of the inflammatory system. In COVID-19 patients, lymphocyte count changes (lymphopenia), along with the level of cytokines, are used as markers of disease severity (Wu and Yang, 2020; Debuc and Smadja, 2021). However, activation of the innate immune system is essential to lead to the production of anti-SARS-CoV-2 antibodies by the adaptive immune system (Moore and June 2020). The inflammatory mediators released in the cytokine storm regulate neutrophil activity and lead to acute respiratory distress syndrome (ARDS). It is suggested that if the normal signals to dampen inflammation are lost, such in COVID-19, a signaling loop between NETs and macrophage can lead to a deregulated inflammation (Lachowicz-Scroggins et al., 2019). IL-1β is secreted by macrophage under the stimulation of NETs (Meher et al., 2018). Previous reports demonstrated that neutrophils treated with IL-1β entered into NETosis (Mitroulis et al., 2011). CXCL-8, another important cytokine, was also reported to be a significant determinant of the NETosis. CXCL-8 had 2 receptors, C-X-C chemokine receptor 1 (CXCR1) and CXCR2, these receptors are involved in various inflammatory disorders such as rheumatoid arthritis or chronic obstructive pulmonary disease. Zhujun A. *et al.* reported that CXCL-8, via its CXCR2 receptor, is able to trigger NETosis. This stimulation of NETosis is done by the G protein receptor kinase pathway (Scr and MAP kinase) and the arrestin pathway. In turn, NETs are able to induce CXCL-8 production by macrophages via the TLR9/NF-κB pathway (Abrams et al., 2019).

In a study of patients with COVID-19, there was a significant difference in plasma NETs levels, COVID-19 patients having a higher level than the controls (control plasma from pre-COVID-19 samples). To evaluate NETs levels in blood, researchers used three well-established markers, namely DNA-free, MPO-DNA and citrullinated histone H3 (Cit-H3) (Zuo et al., 2020). Among these three markers, DNA-free seems to be the less specific for NETs, as it is correlated to the overall level of inflammation of the patient (Zuo et al., 2020). In addition to the increase of these biomarkers, it seems that SARS-CoV-2 patient’s plasma is also an activator of NETosis (Zuo et al., 2020). Taken together, these elements lead to the hypothesis that severe COVID-19 is accompanied with a pro-NETosis state (Zuo et al., 2020). It should be mentioned that Cit-H3 levels are not always correlated with other markers but are more associated to platelet counts. The formation of Cit-H3 seems to be closely related to the activity of PAD4. However, PAD4 is not always involved in the same way in the NETosis process. When NETosis is activated via the formation of ROS, PAD4 is not an important pathway. This difference explains the disparity between cases in regard to Cit-H3 levels and it suggests that there are multiple pathways involved in COVID-19 NETosis (Zuo et al., 2020). Indeed, it has been shown that NETs correlated with IL-1β and IL-6 levels in pulmonary pathologies or in cases of deep vein thrombosis (Zuo et al., 2020). It should be added that in some cases the infection of neutrophils by viruses such as SARS-CoV-2 could directly activate the formation of NETs. This is sustained by the fact that neutrophils, when exposed to the live SARS-CoV-2, developed NETs to a greater extent than other neutrophils. (Veras et al., 2020). One of the characteristics of SARS-CoV-2 is its ability to bind to the human angiotensin converting enzyme receptor (hACE2). The virus also uses the transmembrane protease serine 2 (TMPRSS2) for spike protein priming (Rahman et al., 2020). This pathway could also be involved in the mechanism by which SARS-CoV-2 induces NETosis. In the presence of a neutralizing anti-hACE2 antibody or a TMPRSS2 inhibitor, like bromhexine which has not demonstrated any difference in terms of efficacy with placebo according to a clinical trial (Veras et al., 2020), the ability of the virus to induce the release of NETs was abrogated. However, these products do not block other NETosis activation pathways such as phorbol myristate acetate (PMA) (Hussman, 2020; Li et al., 2020; Veras et al., 2020). Neutrophil overactivation with NETs formation can lead to ARDS which is one of the central components of the complications of COVID-19. This is in line with studies reporting that an excessive NETs formation is linked with ARDS and it was found that plasma levels of NETs were higher in patients with ARDS than in patients without ARDS (Caudrillier et al., 2012). In fact, neutrophils from patients with ARDS seem to be predisposed to form NETs (Lefrançais et al., 2018). Nevertheless, whether we considered the predisposition of neutrophils to form NETs or the level of NETs in plasma, both observations are correlated with the severity of ARDS and the resulting mortality (Ebrahimi et al., 2018; Grégoire et al., 2018; Lefrançais et al., 2018). The inflammatory system is therefore the first element to be affected by an infection with SARS-CoV-2 and the deregulation of the inflammatory system leads to most of the known complications of COVID-19.

#### Potential Therapies for COVID-19 Acting on the Inflammatory System

**FIGURE 1:**
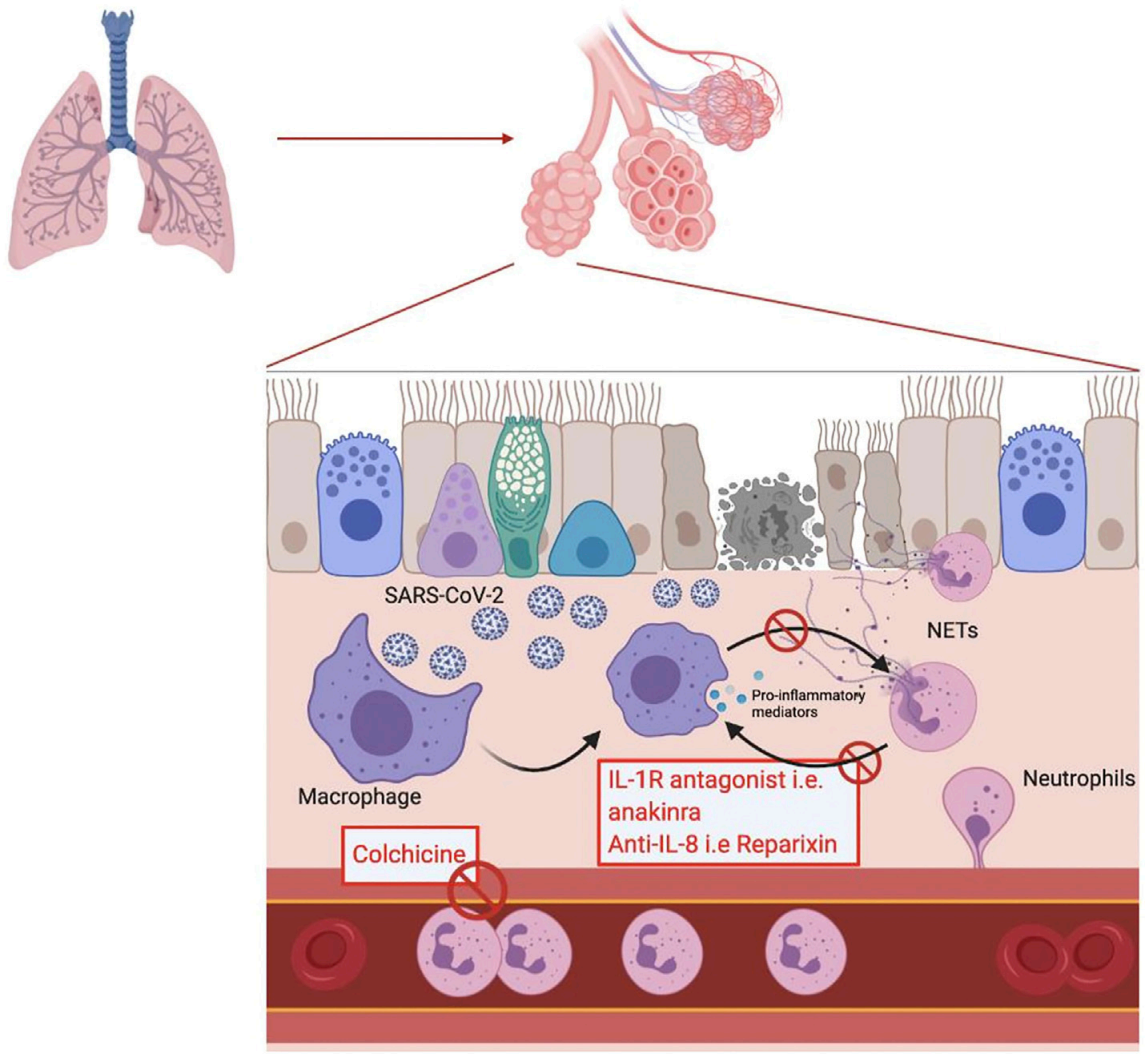
Schematic representation of the inflammatory response to a SARS-CoV-2 viral infection in the alveolar endothelium. In the red box are mentioned the potential treatments acting by inhibiting the mechanisms involved in the response.

Targeting NETosis would therefore be of interest to prevent the occurrence of COVID-19 complications. Several compounds targeting NETosis already exist and others are still under development (Zuo et al., 2020). The targets used by these drugs include the key proteins in NETosis such as NE, PAD4 or Gasdermin G. For example, the irreversible inhibitor of PAD4, Cl-amidine, prevents NETosis by blocking chromatin decondensation. Cl-amidine was initially developed to treat severe inflammatory diseases where NETs are considered as one of the main pathophysiological factors such as in COVID-19 (Yost et al., 2016). There are also NE inhibitors, the oldest being sivelestat, which is already indicated for the treatment of ARDS in Asia since 1998 (Tagami et al., 2014). New NE inhibitors are currently under development such as lonodelestat, alvelestat, elafin or CHF6333 (Barnes et al., 2020). Disulfiram is a drug commonly used in the prevention of alcoholic relapse. Hu J.J. *et al.* have discovered that disulfiram is able to inhibit Gasdermin G which reduces the formation of NETs. A significant reduction in mortality in a mouse model of pneumonia treated with disulfiram vs placebo was demonstrated (Barnes et al., 2020). Another example of a drug that already exists but can be indicated in a physiopathology involving neutrophils is colchicine. Colchicine is usually used to treat gout due to its antimitotic properties, preventing the diapedesis of inflammatory cells and thus the recruitment of neutrophils at the site of inflammation (Barnes et al., 2020). Colchicine also has the ability to inhibit the secretion of IL-1β (Figure 1).

**FIGURE 2:**
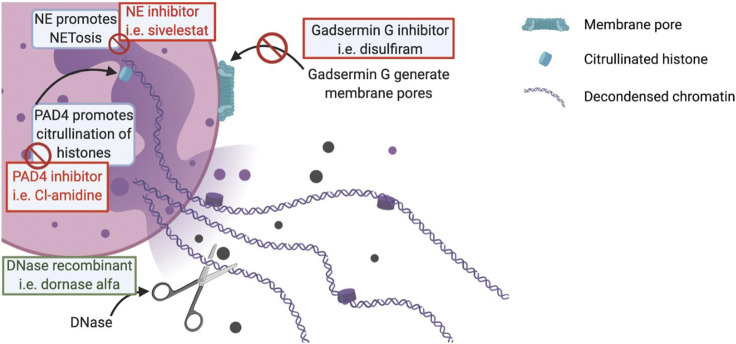
Schematic representation of a NET. In the red boxes are mentioned the potential treatments acting by inhibition of the key mechanisms in the formation of NETs. In the green boxes are mentioned the potential treatments acting by stimulation of different beneficial mechanisms in the context of a viral infection.

The degradation of NETs may also represent an attractive pathway to investigate in order to reduce NETs’ burden. DNases are extremely important for the degradation and the elimination of NETs products. Dornase alfa is a recombinant DNase 1 used as a mucolytic agent in the treatment and management of cystic fibrosis (CF) in conjunction with standard therapies (Yang and Montgomery, 2018). DNase 1 can interact with actin, creating an actin-DNase inactive complex. The reason for this interaction is still unclear, but it could be a form of DNase storage. However, it reduces the activity of DNase 1 analogs such as dornase alpha (Samejima and Earnshaw, 2005). An actin-resistant derivative of dornase alfa, alidornase alfa, is under development, which would allow greater efficacy thanks to its properties to resist to the inhibiting properties of globular actin (Figure 2).

In addition to therapies directly targeting the formation of NETs or their degradation, it is also possible to target the different pathways involved in the amplification loops with NETs. As already mentioned, IL-1β is integrated into an amplification loop with neutrophils, leading to sustained NETosis. Anakinra, an IL-1 receptor antagonist, has been shown to reduce the formation of NETs in studies of pyogenic arthritis, pyoderma gangrenosum and acne (PAPA) syndrome (Mistry et al., 2018). Anakinra is currently being evaluated for its possible use in the treatment of COVID-19. As canakinumab and rilonacept are also IL-1 receptor antagonists, they are also being evaluated in COVID-19 (Figure 1).

In addition to IL-1, CXCL-8 is also involved in an amplification loop with macrophages. BMS-986253 is a monoclonal anti-CXCL-8 antibody and is currently being studied for its potential impact on COVID-19 Similarly, reparixin, a CXCR1 and CXCR2 receptor antagonist, is currently in clinical trials for its use in COVID-19 (Figure 1). (Alfaro et al., 2016; Abrams et al., 2019)

IL-6 is also known to be a key element in the cytokine storm observed in severe COVID-19 patients. Several studies have therefore tested products able to inhibit the IL-6 pathway. These include direct IL-6 inhibitors such as siltuximab or IL-6 receptor antagonists such as tocilizumab and sarilumab. Of note, tocilizumab has been approved in China for use in COVID-19 patients with severe pneumonia. Tocilizumab reduces the likelihood of using mechanical ventilation but does not increase survival (Salama et al., 2021). Sarilumab is an alternative to tocilizumab and is initially indicated for the treatment of rheumatoid arthritis. It is used off label in COVID-19 (Gremese et al., 2020). A recent meta-analysis reported an overall effect in favor of the use of anti-IL-6 for the control of COVID-19. Taken together, these compounds were found to be effective in reducing both mortality and the need for mechanical ventilation (WHO REACT Working Group et al., 2021).

JAK inhibitors such as tofacitinib, baricitinib and ruxolitinib have also been identified as possible therapeutic agents for reducing the burden of severe COVID-19 in patients. The JAK family consists of JAK1, JAK2, JAK3, and tyrosine kinase 2 (TYK2), and the different JAK inhibitors are targeted against one or more of these JAK members. Tofacitinib, which inhibit JAK1, JAK2, and JAK3, and baricitinib and ruxolitinib, both acting against JAK1 and JAK2 are currently under investigation for COVID-19 (Kalil et al., 2021). Recently, it has been shown that baricitinib, at therapeutic doses, has a dual action, including the inhibition of cytokine release, and, through its high affinity for AP2-associated protein kinase 1 (AAK1), which is an important endocytosis regulator, may also inhibit viral cell entry. Such affinity for AAK1 was not seen for tofacitinib and ruxolitinib (Cantini et al., 2020). The clinical trials for the above-mentioned treatments are listed in Table 1.

**TABLE 1:** Table of different clinical trials on the mentioned treatments.

Name of the trial	ClinicalTrials.gov identifier	Disease or condition	Intervention/Treatment	Phase	Estimated enrollment
DISulfiram for Covid-10 (DISCO) Trial (DISCO)	NCT04485130	COVID-19	Disulfiram	2	60
	—	—	Placebo	—	—
Clinical Study to Evaluate the Effects of Disulfiram in Patient With Moderate COVID-19	NCT04594343	COVID-19	Disulfiram	2	200
	—	—	Placebo	—	—
Dornase Alpha for the Treatment of COVID-19	NCT04432987	COVID-19	Pulmozyme	2	60
Efficacy and Safty of aerolizeddornase Alfa Administration in Patient With COVID-19 Induced ARDS (COVIDORNASE)	NCT04355364	COVID-19	Pulmozyme	3	100
	—	—	Standard Procedure	—	—
Nebulised dornase Alfa for Treatment of COVID-19 (COVASE)	NCT04359654	COVID-19	Dornase Alfa Inhalation Solution	2	50
	—	Hypoxia	—	—	—
Pulmozyme to Improve COVID-19 ARDS Outcomes	NCT04402944	COVID-19	Pulmozyme	2	60
	—	—	Placebo	—	—
Dornase Alfa Administered Patients With COVID-19 (DACOVID)	NCT04387786	COVID-19	Dornase Alfa	Observational model	5
	—	Mechanical Ventilation	—	—	—
Phase 2 Trials Using rhDNase to Reduce Mortality in COVID-19 Patients With Respiratory Failure (DAMPENCOVID)	NCT04445285	COVID-19	Pulmozyme/Recombinant human deoxyribonuclease	2	44
	—	—	0.9% sodium chloride	—	—
Dornase Alfa for ARDS in Patients With Severe Acute Respiratory Syndrome-Coronavirus-2 (SARS-CoV-2) (DORNASESARS2)	NCT04402970	SARS-CoV-2	Dornase Alfa Inhalation Solution	3	30
	—	ARDS	—	—	—
Colchicine in Moderate Symptomatic COVID-19 Patients (COLCOVIDBD)	NCT04527562	COVID-19	Colchicine	Not Applicable	299
	—	—	Placebo	—	—
The Greek Study in the Effects of Colchicine in Covid-19 complications Prevention (GRECCO-19)	NCT04326790	COVID-19	Colchicine	2	180
	—	—	Standard treatment	—	—
Colchicine Coronavirus SARS-CoV2 Trial (COLCORONA) (COVID-19)	NCT04322682	COVID-19	Colchicine	3	4,506
	—	—	Placebo oral tablet	—	—
Colchicine in Moderate-severe Hospitalized Patients Before ARDS to Treat COVID-19 (COMBATCOVID19)	NCT04363437	COVID-19	Colchicine	2	70
	—	—	Usual Care	—	—
Study to Investigate the Treatment Effect of Colchicine in Patients With COVID-19	NCT04667780	COVID-19	Colchicine	3	102
	—	—	Standard COVID-19 care	—	—
Effectiveness of Colchicine Among Patients With COVID-19 Infection	NCT04867226	COVID-19	Colchicine 0.5 mg	2	100
	—	—	Usual care treatment	—	—
Anakinra in the Management of COVID-19 Infection	NCT04643678	Covid19	Anakinra	2—3	80
	—	Pneumonia	Standard of Care	—	—
	—	Cytokine release Syndrome	—	—	—
	—	Corona Virus Infection	—	—	—
	—	Viral Infection	—	—	—
Clinical Trial of the Use of Anakinra in Cytokine Storm Syndrome Secondary to Covid-19 (ANA-COVID-GEAS) (ANA-COVID-GEAS)	NCT04443881	COVID-19 Pneumonia	Anakinra 149 mg/ml Prefilled Syringe	2—3	180
Anakinra, COVID-19, Cytokine Storm (SOBI)	NCT04603742	COVID-19	Anakinra	2	170
	—	Cytokine Storm	0.9% Saline	—	—
	—	Mechanical Ventilation Complication	—	—	—
suPAR-Guided Anakinra Treatment for Management of Severe Respiratory Failure by COVID-19 (SAVE-MORE)	NCT04680949	COVID-19	Anakinra	3	606
	—	—	Placebo	—	—
SCIL-1Ra in COVID-19 Feasibility and PK/PD (SCIL_COV19)	NCT04462757	COVID-19	Anakinra 100Mg/0.67MI Inj Syringe	2	5
Anakinra for COVID_19 Respiratory Symptoms (ANACONDA)	NCT04364009	COVID-19	Anakinra plus oSOC	3	71
	—	ANAKINRA Treatment	oSOC	—	—
	—	Optimized Standard of Care (oSOC)	—	—	—
Canakinumab in Covid-19 Cardiac Injury (The Three C Study)	NCT04365153	COVID-19	Canakinumab Injection 600 mg	2	45
	—	—	Canakinumab Injection 300 mg	—	—
	—	—	Placebos	—	—
Study of Efficacy and Safety of canakinumab Treatment for CRS in Participants With COVID-19-induced Pneumonia (CAN-COVID)	NCT04362813	Pneumonia and Cytokine release Syndrome (Covid-19)	Canakinumab	3	451
	—	—	Placebo	—	—
Observational Study, Use of canakinumab Administered Subcutaneously in the Treatment COVID-19 Pneumonia	NCT04348448	COVID-19	Canakinumab 150 mg/ml	Observational Study	100
Canakinumab in Patients With COVID-19 and Type 2 Diabetes (CanCovDia)	NCT04510493	Coronavirus Infection	Canakinumab	3	116
	—	Diabetes Mellitus, Type 2	Placebo	—	—
Anti-Interleukin-8 (Anti-IL-8) for Patients With COVID-19	NCT04347226	Solid Tumor	BMS-986253	2	138
	—	SARS-CoV-2	—	—	—
	—	Hematological Malignancy	—	—	—
Efficacy of Tocilizumab on Patients With COVID-19	NCT04356937	COVID-19	Tocilizumab	3	243
	—	—	Placebo	—	—
Tocilizumab in COVID-19 Pneumonia (TOCIVID-19) (TOCIVID-19)	NCT04317092	COVID-19	Tocilizumab injection	2	402
A Study to Evaluate the Safety and Efficacy of Tocilizumab in Patients With Severe COVID-19 Pneumonia (COVACTA)	NCT04320615	COVID-19	Tocilizumab	3	450
	—	—	Placebo	—	
Sarilumab COVID-19	NCT04327388	COVID-19	Sarilumab SAR153191	3	420
	—	—	Placebo	—	
1912Evaluation of the Effic120acy and Safety of Sariluma220b in Hospitalized Patients With COVID-19	NCT04315298	COVID-19	Sarilumab	2—3	1912
	—	—	Placebo	—	—
Clinical Trial of Sarilumab in Adults With COVID-19 (SARICOR)	NCT04357860	COVID-19	Sarilumab 200 MG/1.14 ML Subcutaneous Solution	2	120
	—	—	Sarilumab 400 MG/2.28 ML Subcutaneous Solution	—	—
	—	—	Best available treatment	—	—
Study on the Use od Sarilumab in Patients With COVID_19 Infection	NCT04386239	COVID-19	Sarilumab Prefilled Syringe	Early phase 1	40
An Observational Study of the Use of Siltuximab (SYLVANT) in Patients Diagnosed With COVID-19 Infection Who Have Developed Serious Respiratory Complications (SISCO)	NCT04322188	Severe Acute Respiratory Syndrome (ARDS) Secondary to SARS-COV-2 Infection	Siltuximab	Observational study	220
Siltuximab in Viral Ards (SILVAR) Study (SILVAR)	NCT04616586	Acute Respiratory	Siltuximab	3	555
	—	Distress Syndrome	Normal Saline	—	—
	—	Lung Diseases	—	—	—
	—	Pneumonia	—	—	—
	—	Respiratory Tract Infections	—	—	—
	—	Respiratory Tract disease	—	—	—
Efficacy and Safety of Siltuximab vs Corticosteroids in Hospitalized Patients With COVID-19 Pneumonia	NCT04329650	COVID-19	Siltuximab	2	2000
	—	—	Methylprednisolone	—	—
Tofacitinib in Hospitalized Patients With COVID-19 Pneumonia	NCT04469114	COVID-19	Tofacitinib 10 mg	2	260
	—	—	Placebo	—	—
Tofacitinib for Treatment of Moderate COVID-19 (I-TOMIC)	NCT04415151	COVID-19	Tofacitinib 10 mg	2	60
	—	—	Placebo	—	—
Efficacy and Safety of Tofacitinib in Patients Wuth COVID-19 Pneumonia (TOFA-COV-2)	NCT04750317	COVID-19	Tofacitinib	2	414
A Study of baricitinib (LY3009104) in Participants With COVID-19 (COV-BARRIER)	NCT04421027	COVID-19	Baricitinib	3	1,400
	—	—	Placebo	—	—
Adaptive COVID-19 Treatment Trial 2 (ACTT-2)	NCT04401579	COVID-19	Remdesivir	3	1,034
	—	—	Baricitinib	—	—
	—	—	Placebo	—	—
Adaptive COVID-19 Treatment Trial 4 (ACTT-4)	NCT04640168	COVID-19	Baricitinib	3	1,500
	—	—	Dexamethasone	—	—
	—	—	Placebo	—	—
	—	—	Remdesivir	—	—
Baricitinib Compared to Standard Therapy in Patients With COVID-19 (BARICIVID-19)	NCT04393051	COVID-19	Baricitinib Oral Tablet	2	126
Baricitinib in Symptomatic Patients Infected by COVID-19: an Open-label, Pilot Study. (BARI-COVID)	NCT04320277	Pharmacological Action in COVID-19	Baricitinib	2—3	200
Ruxolitinib in Covid-19 Patients With Defined Hyperinflammation (RuxCoFlam)	NCT04338958	COVID-19	Ruxolitinib	2	200
Treatment of SARS Caused by COVID-19 With ruxolitinib	NCT04334044	COVID-19	Ruxolitinib	1—2	20
Ruxolitinib in the Treatment of Covid-19	NCT04414098	COVID-19	Ruxolitinib	2	100
Study to Assess the Efficacy and Safety of ruxolitinib in Patients With COVID-19 Associated Cytokine Storm (RUXCOVID)	NCT04362137	Cytokine Storm in COVID-19	Ruxolitinib	3	432
	—	—	Placebo	—	—

The main parenteral anticoagulant used in the treatment and prophylaxis of thrombotic events in COVID-19 is low molecular weight heparin (LMWH) and in some severe cases admitted to ICU, unfractionated heparin (UFH) (Lindahl and Li, 2020). In addition to its anticoagulant properties, LMWH also possesses antiviral and anti-inflammatory activities (Gozzo et al., 2020).

Heparin may reduce P-selectin expression reducing the recruitment of neutrophils at the inflammatory site. Heparin is able to inhibit cathepsin G and NE, which are important inflammatory promoters as outlined in cystic fibrosis and ARDS (Tichelaar et al., 2012; Thachil, 2020). They also have the ability to interact with the vascular endothelium, reducing the expression of several pro-inflammatory factors generated by these cells, including TNF-alpha, IL-6, CXCL-8 and IL-1β. Studies have also demonstrated their ability to reduce RAGE activation thanks to their interaction with the CD11b protein (Gonzales et al., 2014). Finally, heparins have indirect anti-inflammatory effect via their anticoagulant properties. Indeed, a reduction in thrombin levels will lead to a decrease in certain pro-inflammatory proteins such as ICAM-1 and VCAM-1 (Gonzales et al., 2014; Thachil, 2020). These anticoagulant effects, combined with their direct anti-inflammatory effects, make heparin useful in the treatment of COVID-19-related complications.

#### Clinical Evidence of Targeting NETosis in COVID-19

A significant number of clinical trials are underway to evaluate potential therapies targeting NETs in COVID-19. Disulfiram is investigated in two studies for which no results are currently available (Table 1). However, as discussed above, it may be associated with a reduced risk of developing COVID-19, even if this remains to be investigated (Fillmore et al., 2021).

Of the seven studies investigating the use of dornase alfa in the treatment of COVID-19, 2 have completed patient enrollment (NCT04387786 and NCT04402970). The results for these two clinical trials are not yet available but it should be noted that NCT04387786 is an observational study involving only five patients, so the results should be interpreted with caution (Table 1). Several results on the use of dornase alfa in COVID-19 have appeared in the literature. Weber et al. showed efficacy of dornase alfa in a case series of five patients with COVID-19. Toma et al. came to the same conclusion in a study of 39 COVID-19 patients (Weber et al., 2020; Toma et al., 2021).

Of the colchicine studies, five out of the six clinical trials listed in Table 1 have completed recruitment but results are not yet available. Only the NCT04322682 is still recruiting patients. However, short series published in the literature revealed that colchicine has not demonstrated any relevant effect in the treatment of COVID-19 (Table 1) (Cumhur Cure et al., 2020; Reyes et al., 2020).

Regarding the clinical trials investigating the potential effect of anakinra, NCT04680949 is still active but no longer recruiting patients. NCT04443881 has completed recruitment but results are not yet available. NCT04462757 has been terminated due to a lack of patients in the target population of the study. Finally, the clinical trial NCT04364009 was stopped prematurely due to efficacy and patient safety issues (Table 1). Several case series report that the use of anakinra may be beneficial in the control of COVID-19 with a reduction in mortality and the need of mechanical ventilation (Dimopoulos et al., 2020; Navarro-Millán et al., 2020).

Of the canakinumab studies, only NCT04365153 and NCT04362813 have completed recruitment (Table 1).

The study on BMS-986253 is still under recruitment.

Of the three tocilizumab clinical trials mentioned in Table 1, none of them are currently recruiting patients, NCT04356937 and NCT04320615 have completed their recruitment, the TOCIVID-19 study is active but not currently recruiting. Two of the four sarilumab clinical trials have completed enrollment (NCT04315298 and NCT04327388), the other two are still in enrollment (NCT04357860 and NCT04386239). Of the three studies investigating the use of siltuximab in the control of COVID-19, NCT04329650 is still under recruitment and NCT04322188 has completed recruitment. As for NCT04616586, the study has been completed and concluded that the REMAP-CAP and RECOVERY sub-study results appear to support the survival benefit of tocilizumab in corticosteroid-treated or untreated patients with critical COVID-19-associated ARDS (Table 1). Regarding the use of anti-IL-6 in COVID-19, a meta-analysis showed a favorable effect on mortality at 28 days after randomization. Indeed, 1,407 out of 6,449 people died in the tocilizumab group versus 1,158 out of 4,481 in the placebo group. This represents an absolute mortality risk of 22% for the anti-IL-6 group and 25% for the placebo group. Beneficial effects on the use of assisted ventilation were also demonstrated (WHO REACT Working Group et al., 2021).

Only study NCT04415151 with tofacitinib is still enrolling, the other two studies have completed recruitment. Concerning the baricitinib studies, only NCT04401579 and NCT04421027 have finished their recruitment, the other studies are still under recruitment. Concerning the ruxolitinib studies, only NCT04338958 is still under recruitment, the others have completed their recruitment (Table 1). The JAK inhibitors baricitinib, ruxolitinib and tofacitinib have been shown to be well tolerated in patients (Hoang et al., 2021). A study of patients hospitalised with COVID-19 pneumonia showed that there was a reduction in 30-days mortality when treated with baricitinib. This study showed an absolute risk reduction of 18.5% in the population aged over 70 years. These results would be in agreement with the unpublished results of the COV-BARRIER study (NCT04421027) (Table 1) where a 38% reduction in 28-days mortality has been observed (Abizanda et al., 2021).

## Conclusion

This review has highlighted the importance of NETs in a pathology such as COVID-19 although their impact was already known in some autoimmune diseases such as systemic lupus erythematosus. The pathophysiology of COVID-19 is complex and involves different systems including the complement, the inflammatory and the coagulation systems, a well-known triad in ARDS. All of these systems interact with each other with the starting point being the deregulation of the inflammatory system and the appearance of the cytokine storm. This leads to multisystem failure, especially in the case of disseminated intravascular coagulation and lung disorders. The management of hospitalized COVID-19 patients is challenging and may require drugs acting on different pathways to minimize the burden of the disease, especially in the more severe cases. Therapies acting on NETs may play an important role, as it is the cornerstone of many subsequent complications. This review focuses only on the NETosis aspect of the triad involved in COVID-19. This is due to the increasing importance of the involvement of NETs in the disease. This aspect is nevertheless indistinguishable from the other elements with which NETs interact. As mentioned, several treatments targeting NETs are currently being evaluated, at various stages of development. Some of these treatments already exist for other indications. Although the vaccine strategy is advancing rapidly in many countries, this is not the case worldwide, some countries are still very affected by the disease. The importance of having treatments that can act on the severity of the response to the infection are therefore essential. Moreover, the appearance of different variants could compromise the effectiveness of the different vaccines.
