# Supplementary material for: NETosis and the Immune System in COVID-19: Mechanisms and Potential Treatments
Source: Front Pharmacol. 2021 Aug 5;12:708302. doi: 10.3389/fphar.2021.708302 (PMC8376580; doi:10.3389/fphar.2021.708302)
Supplement: Supplementary file 1 [file Table1.DOCX]

Supplemental data 1:

*Screened articles list*

Review

1. Ackermann M, Anders HJ, Bilyy R, Bowlin GL, Daniel C, De Lorenzo R, et al. Patients with COVID-19: in the dark-NETs of neutrophils. Cell Death Differ. 2021:1-15.

2. Allegra A, Innao V, Allegra AG, Musolino C. Coagulopathy and thromboembolic events in patients with SARS-CoV-2 infection: pathogenesis and management strategies. Ann Hematol. 2020;99(9):1953-65.

3. Bai X, Hippensteel J, Leavitt A, Maloney JP, Beckham D, Garcia C, et al. Hypothesis: Alpha-1-antitrypsin is a promising treatment option for COVID-19. Med Hypotheses. 2021;146:110394.

4. Barnes BJ, Adrover JM, Baxter-Stoltzfus A, Borczuk A, Cools-Lartigue J, Crawford JM, et al. Targeting potential drivers of COVID-19: Neutrophil extracellular traps. J Exp Med. 2020;217(6).

5. Bautista-Becerril B, Campi-Caballero R, Sevilla-Fuentes S, Hernandez-Regino LM, Hanono A, Flores-Bustamante A, et al. Immunothrombosis in COVID-19: Implications of Neutrophil Extracellular Traps. Biomolecules. 2021;11(5).

6. Beltran-Garcia J, Osca-Verdegal R, Pallardo FV, Ferreres J, Rodriguez M, Mulet S, et al. Oxidative Stress and Inflammation in COVID-19-Associated Sepsis: The Potential Role of Anti-Oxidant Therapy in Avoiding Disease Progression. Antioxidants (Basel). 2020;9(10).

7. Borges L, Pithon-Curi TC, Curi R, Hatanaka E. COVID-19 and Neutrophils: The Relationship between Hyperinflammation and Neutrophil Extracellular Traps. Mediators Inflamm. 2020;2020:8829674.

8. Cavalcante-Silva LHA, Carvalho DCM, Lima EA, Galvao J, da Silva JSF, Sales-Neto JM, et al. Neutrophils and COVID-19: The road so far. Int Immunopharmacol. 2021;90:107233.

9. Chen X, Guo H, Qiu L, Zhang C, Deng Q, Leng Q. Immunomodulatory and Antiviral Activity of Metformin and Its Potential Implications in Treating Coronavirus Disease 2019 and Lung Injury. Front Immunol. 2020;11:2056.

10. Chen X, Yu C, Jing H, Wang C, Zhao X, Zhang J, et al. COVID-19 associated thromboinflammation of renal capillary: potential mechanisms and treatment. Am J Transl Res. 2020;12(12):7640-56.

11. Chiang CC, Korinek M, Cheng WJ, Hwang TL. Targeting Neutrophils to Treat Acute Respiratory Distress Syndrome in Coronavirus Disease. Front Pharmacol. 2020;11:572009.

12. Cicco S, Cicco G, Racanelli V, Vacca A. Neutrophil Extracellular Traps (NETs) and Damage-Associated Molecular Patterns (DAMPs): Two Potential Targets for COVID-19 Treatment. Mediators Inflamm. 2020;2020:7527953.

13. Crispin PJ, Montague SJ. Megakaryocytes: Masters of Innate Immunity? Arterioscler Thromb Vasc Biol. 2020;40(12):2812-4.

14. de Buhr N, von Kockritz-Blickwede M. The Balance of Neutrophil Extracellular Trap Formation and Nuclease Degradation: an Unknown Role of Bacterial Coinfections in COVID-19 Patients? mBio. 2021;12(1).

15. Dominguez-Diaz C, Varela-Trinidad GU, Munoz-Sanchez G, Solorzano-Castanedo K, Avila-Arrezola KE, Iniguez-Gutierrez L, et al. To Trap a Pathogen: Neutrophil Extracellular Traps and Their Role in Mucosal Epithelial and Skin Diseases. Cells. 2021;10(6).

16. Dowey R, Iqbal A, Heller SR, Sabroe I, Prince LR. A Bittersweet Response to Infection in Diabetes; Targeting Neutrophils to Modify Inflammation and Improve Host Immunity. Front Immunol. 2021;12:678771.

17. Fei Y, Tang N, Liu H, Cao W. Coagulation Dysfunction. Arch Pathol Lab Med. 2020;144(10):1223-9.

18. Fernandez S, Moreno-Castano AB, Palomo M, Martinez-Sanchez J, Torramade-Moix S, Tellez A, et al. Distinctive Biomarker Features in The Endotheliopathy of COVID-19 and Septic Syndromes. Shock. 2021.

19. Gando S, Wada T. Thromboplasminflammation in COVID-19 Coagulopathy: Three Viewpoints for Diagnostic and Therapeutic Strategies. Front Immunol. 2021;12:649122.

20. Hartog N, Faber W, Frisch A, Bauss J, Bupp CP, Rajasekaran S, et al. SARS-CoV-2 infection: molecular mechanisms of severe outcomes to suggest therapeutics. Expert Rev Proteomics. 2021;18(2):105-18.

21. Hasselbalch HC, Skov V, Kjaer L, Ellervik C, Poulsen A, Poulsen TD, et al. COVID-19 as a mediator of interferon deficiency and hyperinflammation: Rationale for the use of JAK1/2 inhibitors in combination with interferon. Cytokine Growth Factor Rev. 2021;60:28-45.

22. Hazeldine J, Lord JM. Neutrophils and COVID-19: Active Participants and Rational Therapeutic Targets. Front Immunol. 2021;12:680134.

23. Hussman JP. Cellular and Molecular Pathways of COVID-19 and Potential Points of Therapeutic Intervention. Front Pharmacol. 2020;11:1169.

24. Iliadi V, Konstantinidou I, Aftzoglou K, Iliadis S, Konstantinidis TG, Tsigalou C. The Emerging Role of Neutrophils in the Pathogenesis of Thrombosis in COVID-19. Int J Mol Sci. 2021;22(10).

25. Illanes-Alvarez F, Marquez-Ruiz D, Marquez-Coello M, Cuesta-Sancho S, Giron-Gonzalez JA. Similarities and differences between HIV and SARS-CoV-2. Int J Med Sci. 2021;18(3):846-51.

26. Jain A, Lamperti M, Doyle DJ. Dexmedetomidine: another arrow in the quiver to fight COVID-19 in intensive care units. Br J Anaesth. 2021;126(1):e35-e8.

27. Janiuk K, Jablonska E, Garley M. Significance of NETs Formation in COVID-19. Cells. 2021;10(1).

28. Jayarangaiah A, Kariyanna PT, Chen X, Jayarangaiah A, Kumar A. COVID-19-Associated Coagulopathy: An Exacerbated Immunothrombosis Response. Clin Appl Thromb Hemost. 2020;26:1076029620943293.

29. Jemberie WB, Stewart Williams J, Eriksson M, Gronlund AS, Ng N, Blom Nilsson M, et al. Substance Use Disorders and COVID-19: Multi-Faceted Problems Which Require Multi-Pronged Solutions. Front Psychiatry. 2020;11:714.

30. Kalyanaraman B. Do free radical NETwork and oxidative stress disparities in African Americans enhance their vulnerability to SARS-CoV-2 infection and COVID-19 severity? Redox Biol. 2020;37:101721.

31. Kanthi Y, Knight JS, Zuo Y, Pinsky DJ. New (re)purpose for an old drug: purinergic modulation may extinguish the COVID-19 thromboinflammatory firestorm. JCI Insight. 2020;5(14).

32. Khalil BA, Elemam NM, Maghazachi AA. Chemokines and chemokine receptors during COVID-19 infection. Comput Struct Biotechnol J. 2021;19:976-88.

33. Klopf J, Brostjan C, Eilenberg W, Neumayer C. Neutrophil Extracellular Traps and Their Implications in Cardiovascular and Inflammatory Disease. Int J Mol Sci. 2021;22(2).

34. Kvietys PR, Fakhoury HMA, Kadan S, Yaqinuddin A, Al-Mutairy E, Al-Kattan K. COVID-19: Lung-Centric Immunothrombosis. Front Cell Infect Microbiol. 2021;11:679878.

35. Laforge M, Elbim C, Frere C, Hemadi M, Massaad C, Nuss P, et al. Tissue damage from neutrophil-induced oxidative stress in COVID-19. Nat Rev Immunol. 2020;20(9):515-6.

36. Lee CCE, Ali K, Connell D, Mordi IR, George J, Lang EM, et al. COVID-19-Associated Cardiovascular Complications. Diseases. 2021;9(3).

37. Lintzmaier Petiz L, Glaser T, Scharfstein J, Ratajczak MZ, Ulrich H. P2Y14 Receptor as a Target for Neutrophilia Attenuation in Severe COVID-19 Cases: From Hematopoietic Stem Cell Recruitment and Chemotaxis to Thrombo-inflammation. Stem Cell Rev Rep. 2021;17(1):241-52.

38. Makatsariya A, Slukhanchuk E, Bitsadze V, Khizroeva J, Tretyakova M, Tsibizova V, et al. COVID-19, neutrophil extracellular traps and vascular complications in obstetric practice. J Perinat Med. 2020;48(9):985-94.

39. Martinod K, Deppermann C. Immunothrombosis and thromboinflammation in host defense and disease. Platelets. 2021;32(3):314-24.

40. Misra DP, Thomas KN, Gasparyan AY, Zimba O. Mechanisms of thrombosis in ANCA-associated vasculitis. Clin Rheumatol. 2021:1-9.

41. Morris G, Bortolasci CC, Puri BK, Olive L, Marx W, O'Neil A, et al. Preventing the development of severe COVID-19 by modifying immunothrombosis. Life Sci. 2021;264:118617.

42. Mozzini C, Girelli D. The role of Neutrophil Extracellular Traps in Covid-19: Only an hypothesis or a potential new field of research? Thromb Res. 2020;191:26-7.

43. Mulay SR, Anders HJ. Neutrophils and Neutrophil Extracellular Traps Regulate Immune Responses in Health and Disease. Cells. 2020;9(9).

44. Nakazawa D, Ishizu A. Immunothrombosis in severe COVID-19. EBioMedicine. 2020;59:102942.

45. Narasaraju T, Tang BM, Herrmann M, Muller S, Chow VTK, Radic M. Neutrophilia and NETopathy as Key Pathologic Drivers of Progressive Lung Impairment in Patients With COVID-19. Front Pharmacol. 2020;11:870.

46. Nathan C. Neutrophils and COVID-19: Nots, NETs, and knots. J Exp Med. 2020;217(9).

47. Omarjee L, Meilhac O, Perrot F, Janin A, Mahe G. Can Ticagrelor be used to prevent sepsis-induced coagulopathy in COVID-19? Clin Immunol. 2020;216:108468.

48. Pramitasuri TI, Laksmidewi A, Putra IBK, Dalimartha FA. Neutrophil Extracellular Traps in Coronavirus Disease-19-Associated Ischemic Stroke: A Novel Avenue in Neuroscience. Exp Neurobiol. 2021;30(1):1-12.

49. Pujhari S, Paul S, Ahluwalia J, Rasgon JL. Clotting disorder in severe acute respiratory syndrome coronavirus 2. Rev Med Virol. 2021;31(3):e2177.

50. Rodrigues PRS, Alrubayyi A, Pring E, Bart VMT, Jones R, Coveney C, et al. Innate immunology in COVID-19-a living review. Part II: dysregulated inflammation drives immunopathology. Oxf Open Immunol. 2020;1(1):iqaa005.

51. Sabbatini M, Magnelli V, Reno F. NETosis in Wound Healing: When Enough Is Enough. Cells. 2021;10(3).

52. Sawadogo SA, Dighero-Kemp B, Ouedraogo DD, Hensley L, Sakande J. How NETosis could drive "Post-COVID-19 syndrome" among survivors. Immunol Lett. 2020;228:35-7.

53. Schonrich G, Raftery MJ, Samstag Y. Devilishly radical NETwork in COVID-19: Oxidative stress, neutrophil extracellular traps (NETs), and T cell suppression. Adv Biol Regul. 2020;77:100741.

54. Seif S, Ayuna A, Kumar A, Macdonald J. Massive coronary thrombosis caused primary percutaneous coronary intervention to fail in a COVID-19 patient with ST-elevation myocardial infarction. Catheter Cardiovasc Interv. 2021;97(5):E667-E9.

55. Seitz R, Gurtler L, Schramm W. Thromboinflammation in COVID-19: Can alpha2 -macroglobulin help to control the fire? J Thromb Haemost. 2021;19(2):351-4.

56. Shaw RJ, Bradbury C, Abrams ST, Wang G, Toh CH. COVID-19 and Immunothrombosis: emerging understanding and clinical management. Br J Haematol. 2021.

57. Soy M, Keser G, Atagunduz P, Tabak F, Atagunduz I, Kayhan S. Cytokine storm in COVID-19: pathogenesis and overview of anti-inflammatory agents used in treatment. Clin Rheumatol. 2020;39(7):2085-94.

58. Stegelmeier AA, Darzianiazizi M, Hanada K, Sharif S, Wootton SK, Bridle BW, et al. Type I Interferon-Mediated Regulation of Antiviral Capabilities of Neutrophils. Int J Mol Sci. 2021;22(9).

59. Sung PS, Hsieh SL. C-type lectins and extracellular vesicles in virus-induced NETosis. J Biomed Sci. 2021;28(1):46.

60. Teluguakula N. Neutrophils Set Extracellular Traps to Injure Lungs in Coronavirus Disease 2019. J Infect Dis. 2021;223(9):1503-5.

61. Thierry AR. Anti-protease Treatments Targeting Plasmin(ogen) and Neutrophil Elastase May Be Beneficial in Fighting COVID-19. Physiol Rev. 2020;100(4):1597-8.

62. Thierry AR. Does the newly observed inflammatory syndrome in children demonstrate a link between uncontrolled neutrophil extracellular traps formation and COVID-19? Pediatr Res. 2021;89(4):716-7.

63. Thierry AR, Roch B. SARS-CoV2 may evade innate immune response, causing uncontrolled neutrophil extracellular traps formation and multi-organ failure. Clin Sci (Lond). 2020;134(12):1295-300.

64. Thierry AR, Roch B. Neutrophil Extracellular Traps and By-Products Play a Key Role in COVID-19: Pathogenesis, Risk Factors, and Therapy. J Clin Med. 2020;9(9).

65. Toma A, Darwish C, Taylor M, Harlacher J, Darwish R. The Use of Dornase Alfa in the Management of COVID-19-Associated Adult Respiratory Distress Syndrome. Crit Care Res Pract. 2021;2021:8881115.

66. Tomar B, Anders HJ, Desai J, Mulay SR. Neutrophils and Neutrophil Extracellular Traps Drive Necroinflammation in COVID-19. Cells. 2020;9(6).

67. Vorobjeva NV. Neutrophil Extracellular Traps: New Aspects. Moscow Univ Biol Sci Bull. 2020;75(4):173-88.

68. Vorobjeva NV, Chernyak BV. NETosis: Molecular Mechanisms, Role in Physiology and Pathology. Biochemistry (Mosc). 2020;85(10):1178-90.

69. Wang J, Jiang M, Chen X, Montaner LJ. Cytokine storm and leukocyte changes in mild versus severe SARS-CoV-2 infection: Review of 3939 COVID-19 patients in China and emerging pathogenesis and therapy concepts. J Leukoc Biol. 2020;108(1):17-41.

70. Yang J, Wu Z, Long Q, Huang J, Hong T, Liu W, et al. Insights Into Immunothrombosis: The Interplay Among Neutrophil Extracellular Trap, von Willebrand Factor, and ADAMTS13. Front Immunol. 2020;11:610696.

71. Yang SC, Tsai YF, Pan YL, Hwang TL. Understanding the role of neutrophils in acute respiratory distress syndrome. Biomed J. 2020.

72. Yaqinuddin A, Kvietys P, Kashir J. COVID-19: Role of neutrophil extracellular traps in acute lung injury. Respir Investig. 2020;58(5):419-20.

73. Zuo Y, Kanthi Y, Knight JS, Kim AHJ. The interplay between neutrophils, complement, and microthrombi in COVID-19. Best Pract Res Clin Rheumatol. 2021;35(1):101661.

Ex-vivo investigations

74. Zuo Y, Zuo M, Yalavarthi S, Gockman K, Madison JA, Shi H, et al. Neutrophil extracellular traps and thrombosis in COVID-19. J Thromb Thrombolysis. 2021;51(2):446-53.

75. Zuo Y, Zuo M, Yalavarthi S, Gockman K, Madison JA, Shi H, et al. Neutrophil extracellular traps and thrombosis in COVID-19. medRxiv. 2020.

76. Zuo Y, Yalavarthi S, Shi H, Gockman K, Zuo M, Madison JA, et al. Neutrophil extracellular traps (NETs) as markers of disease severity in COVID-19. medRxiv. 2020.

77. Zuo Y, Yalavarthi S, Shi H, Gockman K, Zuo M, Madison JA, et al. Neutrophil extracellular traps in COVID-19. JCI Insight. 2020;5(11).

78. Zuo Y, Yalavarthi S, Navaz SA, Hoy CK, Harbaugh A, Gockman K, et al. Autoantibodies stabilize neutrophil extracellular traps in COVID-19. JCI Insight. 2021.

79. Zuo Y, Estes SK, Gandhi AA, Yalavarthi S, Ali RA, Shi H, et al. Prothrombotic antiphospholipid antibodies in COVID-19. medRxiv. 2020.

80. Zhang Y, Han K, Du C, Li R, Liu J, Zeng H, et al. Carboxypeptidase B blocks ex vivo activation of the anaphylatoxin-neutrophil extracellular trap axis in neutrophils from COVID-19 patients. Crit Care. 2021;25(1):51.

81. Weber AG, Chau AS, Egeblad M, Barnes BJ, Janowitz T. Nebulized in-line endotracheal dornase alfa and albuterol administered to mechanically ventilated COVID-19 patients: a case series. Mol Med. 2020;26(1):91.

82. Wang J, Li Q, Yin Y, Zhang Y, Cao Y, Lin X, et al. Excessive Neutrophils and Neutrophil Extracellular Traps in COVID-19. Front Immunol. 2020;11:2063.

83. Vorobjeva NV, Sud'ina GF, Chernyak BV. Mitochondria Are Potential Targets for the Development of New Drugs Against Neutrophilic Inflammation in Severe Pneumonia Including COVID-19. Front Pharmacol. 2021;12:609508.

84. Veras FP, Pontelli MC, Silva CM, Toller-Kawahisa JE, de Lima M, Nascimento DC, et al. SARS-CoV-2-triggered neutrophil extracellular traps mediate COVID-19 pathology. J Exp Med. 2020;217(12).

85. Tumminello G, Barbieri L, Lucreziotti S, Gentile D, Conconi B, Centola M, et al. Impact of COVID-19 on STEMI: Second youth for fibrinolysis or time to centralized approach? Int J Cardiol Heart Vasc. 2020;30:100600.

86. Strich JR, Ramos-Benitez MJ, Randazzo D, Stein SR, Babyak A, Davey RT, et al. Fostamatinib Inhibits Neutrophils Extracellular Traps Induced by COVID-19 Patient Plasma: A Potential Therapeutic. J Infect Dis. 2021;223(6):981-4.

87. Staats LAN, Pfeiffer H, Knopf J, Lindemann A, Furst J, Kremer AE, et al. IgA2 Antibodies against SARS-CoV-2 Correlate with NET Formation and Fatal Outcome in Severely Diseased COVID-19 Patients. Cells. 2020;9(12).

88. Skendros P, Mitsios A, Chrysanthopoulou A, Mastellos DC, Metallidis S, Rafailidis P, et al. Complement and tissue factor-enriched neutrophil extracellular traps are key drivers in COVID-19 immunothrombosis. J Clin Invest. 2020;130(11):6151-7.

89. Shi H, Zuo Y, Yalavarthi S, Gockman K, Zuo M, Madison JA, et al. Neutrophil calprotectin identifies severe pulmonary disease in COVID-19. J Leukoc Biol. 2021;109(1):67-72.

90. Seery V, Raiden SC, Algieri SC, Grisolia NA, Filippo D, De Carli N, et al. Blood neutrophils from children with COVID-19 exhibit both inflammatory and anti-inflammatory markers. EBioMedicine. 2021;67:103357.

91. Schurink B, Roos E, Radonic T, Barbe E, Bouman CSC, de Boer HH, et al. Viral presence and immunopathology in patients with lethal COVID-19: a prospective autopsy cohort study. Lancet Microbe. 2020;1(7):e290-e9.

92. Radermecker C, Detrembleur N, Guiot J, Cavalier E, Henket M, d'Emal C, et al. Neutrophil extracellular traps infiltrate the lung airway, interstitial, and vascular compartments in severe COVID-19. J Exp Med. 2020;217(12).

93. Petito E, Falcinelli E, Paliani U, Cesari E, Vaudo G, Sebastiano M, et al. Association of Neutrophil Activation, More Than Platelet Activation, With Thrombotic Complications in Coronavirus Disease 2019. J Infect Dis. 2021;223(6):933-44.

94. Park HH, Park W, Lee YY, Kim H, Seo HS, Choi DW, et al. Bioinspired DNase-I-Coated Melanin-Like Nanospheres for Modulation of Infection-Associated NETosis Dysregulation. Adv Sci (Weinh). 2020;7(23):2001940.

95. Park HH, Kim H, Lee HS, Seo EU, Kim JE, Lee JH, et al. PEGylated nanoparticle albumin-bound steroidal ginsenoside derivatives ameliorate SARS-CoV-2-mediated hyper-inflammatory responses. Biomaterials. 2021;273:120827.

96. Parackova Z, Zentsova I, Bloomfield M, Vrabcova P, Smetanova J, Klocperk A, et al. Disharmonic Inflammatory Signatures in COVID-19: Augmented Neutrophils' but Impaired Monocytes' and Dendritic Cells' Responsiveness. Cells. 2020;9(10).

97. Ouwendijk WJD, Raadsen MP, van Kampen JJA, Verdijk RM, von der Thusen JH, Guo L, et al. High Levels of Neutrophil Extracellular Traps Persist in the Lower Respiratory Tract of Critically Ill Patients With Coronavirus Disease 2019. J Infect Dis. 2021;223(9):1512-21.

98. Ondracek AS, Lang IM. Neutrophil Extracellular Traps as Prognostic Markers in COVID-19: A Welcome Piece to the Puzzle. Arterioscler Thromb Vasc Biol. 2021;41(2):995-8.

99. Nishibori M, Stonestreet BS. Understanding of COVID-19 Pathology: Much More Attention to Plasma Proteins. Front Immunol. 2021;12:656099.

100. Nicolai L, Leunig A, Brambs S, Kaiser R, Weinberger T, Weigand M, et al. Immunothrombotic Dysregulation in COVID-19 Pneumonia Is Associated With Respiratory Failure and Coagulopathy. Circulation. 2020;142(12):1176-89.

101. Nicolai L, Leunig A, Brambs S, Kaiser R, Joppich M, Hoffknecht ML, et al. Vascular neutrophilic inflammation and immunothrombosis distinguish severe COVID-19 from influenza pneumonia. J Thromb Haemost. 2021;19(2):574-81.

102. Ng H, Havervall S, Rosell A, Aguilera K, Parv K, von Meijenfeldt FA, et al. Circulating Markers of Neutrophil Extracellular Traps Are of Prognostic Value in Patients With COVID-19. Arterioscler Thromb Vasc Biol. 2021;41(2):988-94.

103. Middleton EA, He XY, Denorme F, Campbell RA, Ng D, Salvatore SP, et al. Neutrophil extracellular traps contribute to immunothrombosis in COVID-19 acute respiratory distress syndrome. Blood. 2020;136(10):1169-79.

104. Mastellos DC, Pires da Silva BGP, Fonseca BAL, Fonseca NP, Auxiliadora-Martins M, Mastaglio S, et al. Complement C3 vs C5 inhibition in severe COVID-19: Early clinical findings reveal differential biological efficacy. Clin Immunol. 2020;220:108598.

105. Masso-Silva JA, Moshensky A, Lam MTY, Odish M, Patel A, Xu L, et al. Increased peripheral blood neutrophil activation phenotypes and NETosis in critically ill COVID-19 patients: a case series and review of the literature. Clin Infect Dis. 2021.

106. Leppkes M, Knopf J, Naschberger E, Lindemann A, Singh J, Herrmann I, et al. Vascular occlusion by neutrophil extracellular traps in COVID-19. EBioMedicine. 2020;58:102925.

107. Hidalgo A. A NET-thrombosis axis in COVID-19. Blood. 2020;136(10):1118-9.

108. Gueant JL, Gueant-Rodriguez RM, Fromonot J, Oussalah A, Louis H, Chery C, et al. Elastase and exacerbation of neutrophil innate immunity are involved in multi-visceral manifestations of COVID-19. Allergy. 2021;76(6):1846-58.

109. Gueant JL, Fromonot J, Gueant-Rodriguez RM, Lacolley P, Guieu R, Regnault V. Blood myeloperoxidase-DNA, a biomarker of early response to SARS-CoV-2 infection? Allergy. 2021;76(3):892-6.

110. Gremese E, Ferraccioli G. The pathogenesis of microthrombi in COVID-19 cannot be controlled by DOAC: NETosis should be the target. J Intern Med. 2021;289(3):420-1.

111. Godement M, Zhu J, Cerf C, Vieillard-Baron A, Maillon A, Zuber B, et al. Neutrophil Extracellular Traps in SARS-CoV2 Related Pneumonia in ICU Patients: The NETCOV2 Study. Front Med (Lausanne). 2021;8:615984.

112. Formiga RO, Amaral FC, Souza CF, Mendes D, Wanderley CWS, Lorenzini CB, et al. Neuraminidase inhibitors rewire neutrophil function in murine sepsis and COVID-19 patient cells. bioRxiv. 2020.

113. Fisher J, Mohanty T, Karlsson C, Khademi SMH, Malmstrom E, Frigyesi A, et al. Proteome profiling of recombinant DNase therapy in reducing NETs and aiding recovery in COVID-19 patients. Mol Cell Proteomics. 2021:100113.

114. Fernandez-Ruiz R, Paredes JL, Niewold TB. COVID-19 in patients with systemic lupus erythematosus: lessons learned from the inflammatory disease. Transl Res. 2021;232:13-36.

115. Fernandez-Perez MP, Aguila S, Reguilon-Gallego L, de Los Reyes-Garcia AM, Minano A, Bravo-Perez C, et al. Neutrophil extracellular traps and von Willebrand factor are allies that negatively influence COVID-19 outcomes. Clin Transl Med. 2021;11(1):e268.

116. Englert H, Rangaswamy C, Deppermann C, Sperhake JP, Krisp C, Schreier D, et al. Defective NET clearance contributes to sustained FXII activation in COVID-19-associated pulmonary thrombo-inflammation. EBioMedicine. 2021;67:103382.

117. Delaveris CS, Wilk AJ, Riley NM, Stark JC, Yang SS, Rogers AJ, et al. Synthetic Siglec-9 Agonists Inhibit Neutrophil Activation Associated with COVID-19. ACS Cent Sci. 2021;7(4):650-7.

118. Delaveris CS, Wilk AJ, Riley NM, Stark JC, Yang SS, Rogers AJ, et al. Synthetic Siglec-9 Agonists Inhibit Neutrophil Activation Associated with COVID-19. ChemRxiv. 2020.

119. Cerami C, Santi GC, Galandra C, Dodich A, Cappa SF, Vecchi T, et al. Covid-19 Outbreak In Italy: Are We Ready for the Psychosocial and the Economic Crisis? Baseline Findings From the PsyCovid Study. Front Psychiatry. 2020;11:556.

120. Cavalier E, Guiot J, Lechner K, Dutsch A, Eccleston M, Herzog M, et al. Circulating Nucleosomes as Potential Markers to Monitor COVID-19 Disease Progression. Front Mol Biosci. 2021;8:600881.

121. Busch MH, Timmermans S, Nagy M, Visser M, Huckriede J, Aendekerk JP, et al. Neutrophils and Contact Activation of Coagulation as Potential Drivers of COVID-19. Circulation. 2020;142(18):1787-90.

122. Brenner SR. Erythropoietin-induced hemoglobin subunit beta may stimulate innate immune RNA virus pattern recognition, suppress reactive oxygen species, reduce ACE2 viral doorway opening, and neutrophil extracellular traps against COVID-19. J Med Virol. 2021;93(1):180-1.

123. Boeckh-Behrens T, Golkowski D, Ikenberg B, Schlegel J, Protzer U, Schulz C, et al. COVID-19-associated Large Vessel Stroke in a 28-year-old Patient : NETs and Platelets Possible Key Players in Acute Thrombus Formation. Clin Neuroradiol. 2021;31(2):511-4.

124. Blasco A, Coronado MJ, Hernandez-Terciado F, Martin P, Royuela A, Ramil E, et al. Assessment of Neutrophil Extracellular Traps in Coronary Thrombus of a Case Series of Patients With COVID-19 and Myocardial Infarction. JAMA Cardiol. 2020;6(4):1-6.

125. Arcanjo A, Logullo J, Menezes CCB, de Souza Carvalho Giangiarulo TC, Dos Reis MC, de Castro GMM, et al. The emerging role of neutrophil extracellular traps in severe acute respiratory syndrome coronavirus 2 (COVID-19). Sci Rep. 2020;10(1):19630.

126. Akgun E, Tuzuner MB, Sahin B, Kilercik M, Kulah C, Cakiroglu HN, et al. Proteins associated with neutrophil degranulation are upregulated in nasopharyngeal swabs from SARS-CoV-2 patients. PLoS One. 2020;15(10):e0240012.

In-vitro investigations

127. Youn YJ, Lee YB, Kim SH, Jin HK, Bae JS, Hong CW. Nucleocapsid and Spike Proteins of SARS-CoV-2 Drive Neutrophil Extracellular Trap Formation. Immune Netw. 2021;21(2):e16.

128. Yaqinuddin A, Kashir J. Novel therapeutic targets for SARS-CoV-2-induced acute lung injury: Targeting a potential IL-1beta/neutrophil extracellular traps feedback loop. Med Hypotheses. 2020;143:109906.

129. Stacey HD, Golubeva D, Posca A, Ang JC, Novakowski KE, Zahoor MA, et al. IgA potentiates NETosis in response to viral infection. Proc Natl Acad Sci U S A. 2021;118(27).

130. Shi H, Gandhi AA, Smith SA, Chiang D, Yalavarthi S, Ali RA, et al. Endothelium-protective, histone-neutralizing properties of the polyanionic agent defibrotide. medRxiv. 2021.

131. Pandolfi L, Bozzini S, Frangipane V, Percivalle E, De Luigi A, Violatto MB, et al. Neutrophil Extracellular Traps Induce the Epithelial-Mesenchymal Transition: Implications in Post-COVID-19 Fibrosis. Front Immunol. 2021;12:663303.

132. Okur HK, Yalcin K, Tastan C, Demir S, Yurtsever B, Karakus GS, et al. Preliminary report of in vitro and in vivo effectiveness of dornase alfa on SARS-CoV-2 infection. New Microbes New Infect. 2020;37:100756.

133. Niu C, Du Y, Kaltashov IA. Towards better understanding of the heparin role in NETosis: feasibility of using native mass spectrometry to monitor interactions of neutrophil elastase with heparin oligomers. Int J Mass Spectrom. 2021;463.

134. Lv D, Xu Y, Cheng H, Ke Y, Zhang X, Ying K. A novel cell-based assay for dynamically detecting neutrophil extracellular traps-induced lung epithelial injuries. Exp Cell Res. 2020;394(2):112101.

135. Lee YY, Park HH, Park W, Kim H, Jang JG, Hong KS, et al. Long-acting nanoparticulate DNase-1 for effective suppression of SARS-CoV-2-mediated neutrophil activities and cytokine storm. Biomaterials. 2021;267:120389.

136. Ding J, Hostallero DE, El Khili MR, Fonseca GJ, Milette S, Noorah N, et al. A network-informed analysis of SARS-CoV-2 and hemophagocytic lymphohistiocytosis genes' interactions points to Neutrophil extracellular traps as mediators of thrombosis in COVID-19. PLoS Comput Biol. 2021;17(3):e1008810.

137. Colling ME, Kanthi Y. COVID-19-associated coagulopathy: An exploration of mechanisms. Vasc Med. 2020;25(5):471-8.

138. Arisan ED, Uysal-Onganer P, Lange S. Putative Roles for Peptidylarginine Deiminases in COVID-19. Int J Mol Sci. 2020;21(13).

Meta-analysis

139. Elshafei MN, El-Bardissy A, Khalil A, Danjuma M, Mubasher M, Abubeker IY, et al. Colchicine use might be associated with lower mortality in COVID-19 patients: A meta-analysis. Eur J Clin Invest. 2021:e13645.

In-vivo investigations

140. Hoang TN, Pino M, Boddapati AK, Viox EG, Starke CE, Upadhyay AA, et al. Baricitinib treatment resolves lower-airway macrophage inflammation and neutrophil recruitment in SARS-CoV-2-infected rhesus macaques. Cell. 2021;184(2):460-75 e21.

141. Hoang TN, Pino M, Boddapati AK, Viox EG, Starke CE, Upadhyay AA, et al. Baricitinib treatment resolves lower airway inflammation and neutrophil recruitment in SARS-CoV-2-infected rhesus macaques. bioRxiv. 2020.

142. Becker K, Beythien G, de Buhr N, Stanelle-Bertram S, Tuku B, Kouassi NM, et al. Vasculitis and Neutrophil Extracellular Traps in Lungs of Golden Syrian Hamsters With SARS-CoV-2. Front Immunol. 2021;12:640842.

Clinical studies

143. Earhart AP, Holliday ZM, Hofmann HV, Schrum AG. Consideration of dornase alfa for the treatment of severe COVID-19 acute respiratory distress syndrome. New Microbes New Infect. 2020;35:100689.

144. Desilles JP, Gregoire C, Le Cossec C, Lambert J, Mophawe O, Losser MR, et al. Efficacy and safety of aerosolized intra-tracheal dornase alfa administration in patients with SARS-CoV-2-induced acute respiratory distress syndrome (ARDS): a structured summary of a study protocol for a randomised controlled trial. Trials. 2020;21(1):548.
